# Comparison of Machine Learning Algorithms in the Prediction of Hospitalized Patients with Schizophrenia

**DOI:** 10.3390/s22072517

**Published:** 2022-03-25

**Authors:** Susel Góngora Alonso, Gonçalo Marques, Deevyankar Agarwal, Isabel De la Torre Díez, Manuel Franco-Martín

**Affiliations:** 1 Department of Signal Theory and Communications, and Telematics Engineering, University of Valladolid, Paseo de Belén, 15, 47011 Valladolid, Spain; susel.gongora@uva.es; 2 Polytechnic of Coimbra, ESTGOH, Rua General Santos Costa, 3400-124 Oliveira do Hospital, Portugal; goncalosantosmarques@gmail.com; 3 EEE Section, Department of Engineering, Higher College of Technology, Muscat 113, Oman; deevyankar.agarwal@hct.edu.om; 4 Psiquiatry Service, Hospital Zamora, 49021 Zamora, Spain; mfrancom@saludcastillayleon.es

**Keywords:** hospitalization, machine learning algorithms, predictive models, random forest, schizophrenia

## Abstract

New computational methods have emerged through science and technology to support the diagnosis of mental health disorders. Predictive models developed from machine learning algorithms can identify disorders such as schizophrenia and support clinical decision making. This research aims to compare the performance of machine learning algorithms: Decision Tree, AdaBoost, Random Forest, Naïve Bayes, Support Vector Machine, and k-Nearest Neighbor in the prediction of hospitalized patients with schizophrenia. The data set used in the study contains a total of 11,884 electronic admission records corresponding to 6933 patients with various mental health disorders; these records belong to the acute units of 11 public hospitals in a region of Spain. Of the total, 5968 records correspond to patients diagnosed with schizophrenia (3002 patients) and 5916 records correspond to patients with other mental health disorders (3931 patients). The results recommend Random Forest with the best accuracy of 72.7%. Furthermore, this algorithm presents 79.6%, 72.8%, 72.7%, and 72.7% for AUC, precision, F1-Score, and recall, respectively. The results obtained suggest that the use of machine learning algorithms can classify hospitalized patients with schizophrenia in this population and help in the hospital management of this type of disorder, to reduce the costs associated with hospitalization.

## 1. Introduction

Currently, data mining and machine learning techniques allow the exploration and analyzation of data patterns through statistical methods and artificial intelligence [1,2,3]. Researchers can obtain correlations and patterns from large data sets to create new knowledge with the help of machine learning and artificial intelligence [4,5,6].

Psychiatry is a field of medicine that specializes in studying and treating mental, emotional, or behavioral disorders [7,8]. Schizophrenia is a severe and debilitating chronic mental illness causing a high burden and healthcare utilization with the global age-standardized point prevalence of schizophrenia at 0.28%, which means 21 million cases worldwide [9]. The onset of the symptoms and diagnosis used to be during the second and third life decades and is controversial regarding the sex ratio, which is balanced between genders depending on the methodology, but in clinical groups has higher prevalence in men, as was found in the current Spanish study [10]. It is characterized by many different symptoms and signs such as thought disorder, delusions, emotional blunting, hallucinations, changes in volition, as well as cognitive deficits [11,12,13,14]. However, the main phenomenological feature is the variety of symptomatology and lack of a pathognomonic symptom or sign.

People with this disorder have higher rates of morbidity and mortality than the general population (adjusted hazard ratio (aHR) = 3.52). Most deaths are related to physical disorders, mainly metabolic syndrome, and its consequences (stroke, hypertension), and infectious diseases [15,16]. However, suicide is also a relevant cause of death in patients with schizophrenia, reaching 5% of them [17]. Indeed, it is considered an underestimated event because about 25–50% of these patients attempt suicide during their life [18]. The current challenge of schizophrenia is an early diagnosis of the illness and treatment to mitigate the progressive impairment of cognitive, skills, and social function of the natural course of the disease. It will also be useful for preventing suicide and improving comprehensive physical and mental treatment. Diagnostic and monitoring depend mainly on their clinical experience, and decisions are made based on the specific case [19,20].

On the other hand, and considering that schizophrenia patients use multiple healthcare services, with higher hospitalizations rates and longer mean duration, it is a relevant health problem. Indeed, this condition imposes a tremendous economic charge both for patients, their families, and society in general [21,22].

Data analysis and decision making are crucial steps, especially in mental illness [23,24]. Classification algorithms such as Logistic Regression, Decision Tree, Random Forest, AdaBoost, Naïve Bayes, k-Nearest Neighbor (k-NN), and Support Vector Machine (SVM) are used in different studies [25,26,27,28] for the diagnosis of patients with Alzheimer’s, Parkinson’s, and mild cognitive impairment (MCI). The application of machine learning in mental health has allowed the prediction of genetic risk, the identification of candidates for biomarkers, or the exploration of etiological mechanisms [29]. Furthermore, the predictive precision of medical data by classifying people as healthy or not allows the development of new therapeutic and preventive strategies in mental health diseases [30,31,32,33].

In clinical activity, the identification of suicide risk in schizophrenia is very relevant and there are many proposals for its detection. In the study [34], the authors sought to find new available clinical resources that accurately identify the suicide risk in schizophrenia. In the study [35], the authors developed a clinically useful predictive model to identify schizophrenia patients who attempt suicide and those who do not, in a sample of 345 participants. The results showed the best metrics in the support vector classifier model and regularized logistic regression with an accuracy of 67.0% and an area under the ROC curve (AUC) of 0.70 and 0.71, respectively.

In studies such as [36], the authors analyzed a predictive system to preventively diagnose schizophrenia disease using SVM, Random Forest, ANN, and Naïve Bayes. The results showed the highest precision of 90.69% using SVM, Random Forest, and Naïve Bayes. In this work, the authors applied feature selection to understand if it was possible to improve the performance using recursive feature elimination.

In [37], the authors investigated a classification algorithm to predict schizophrenia using combined electroencephalogram (EEG) features, obtaining an average accuracy of 78.24% with the SVM algorithm. This work included a small data set with a total of 68 individuals and feature selection methods, and only SVM was tested and not compared with related machine learning approaches.

The authors of [38] have tested algorithms such as SVM, Naïve Bayes, Random Forest, and gradient boosting to predict schizophrenia patients and healthy controls, using 72 individuals corresponding 48 and 24 to schizophrenia patients and healthy controls, respectively. The experiments showed an accuracy of 58.2% using SVM and 68.6% through Random Forest. In [39], the authors with a sample of 21 features obtained accuracy values of 72.3% for Decision Tree, SVM with 78.7%, k-NN with 76.5%, and Random Forest with 85.1%. According to the authors, about 500 patient medical records were processed as the data set and cross-validation was used.

Machine learning algorithms such as Random Forest and SVM have been applied in the studies [40,41] to discriminate between schizophrenia patients and healthy patients using magnetic resonance imaging (MRI). The results of study [41] showed a high performance rate using 504 features. Random Forest had sensitivity = 87.6% and specificity = 95.9%, and SVM had sensitivity = 89.5% and specificity = 94.5%.

In the study [42], the authors classified patients with schizophrenia from healthy patients using the messenger RNA expression level in peripheral blood. The authors compared machine learning algorithms such as artificial neural networks, extreme gradient boosting, SVM, Decision Tree, and Random Forest. The results showed SVM as the best model with AUC of 0.993, sensitivity = 1000, and specificity = 0.895.

EEG signals are used in multiple studies to classify patterns between healthy and schizophrenia patients [43,44,45], using algorithms such as Random Forest, SVM, kNN, and Multilayer Perceptron (MLP). Other studies used a machine learning approach to predict cognitive function in schizophrenia [46], identify people with schizophrenia through social networks [47,48], and develop a predictive model to identify violence in patients with schizophrenia [49].

In the previous studies [50,51], an analysis of the hospitalization of patients with mental disorders in the region of Castilla and León was carried out, determining schizophrenia as the most prevalent disorder in this population. Therefore, the objective of this paper is to compare the performance of machine learning algorithms: Decision Tree, AdaBoost, Random Forest, Naïve Bayes, k-NN, and SVM in the prediction of hospitalized patients with schizophrenia, and to identify the appropriate classification algorithm for this research line that best classifies patients hospitalized with this disease. The authors have tested the previously mentioned low computational machine learning methods since several researchers have used the same methods and it is critical to compare the performance of the methods to understand if the results converge in this field of research. The authors expect Random Forest to provide the best performance based on the previous research activities of other authors [35,36,37,38,39]. These studies are different regarding the number of features, sample population, and algorithms they use to develop their research.

## 2. Materials and Methods

The purpose of the study is to analyze and compare the performance metrics of machine learning algorithms for the recognition of hospitalized patients with schizophrenia. According to previous studies [27,35,50], we selected six algorithms, such as Random Forest, k-NN, AdaBoost, Naïve Bayes, Decision Tree, and SVM, for the development of our research. The authors of [50] have made an initial review of this research topic. Moreover, by performing a more updated analysis of the literature, the authors find relevant studies of schizophrenia [27,35,36,37,38,39,40,41,42,43,44] that focus their research on these machine learning techniques.

### 2.1. Database

The database used in the study contains patient admission records from 11 public hospitals in Castilla and León, Spain, taking the minimum data set following the national rules for saving hospitalized patients’ data. This database has been approved under the Project “Contribution to the Analysis and Development of Data Mining Techniques and Sources in an Internet of Things (IoT) Environment in the Field of Mental Health” by the Ethics Committee of the University of Valladolid (Ref. PI 20-1780).

Data includes the period from 2005 to 2015. We selected the acute units of the 11 hospitals in the region. The data set used contains a total of 6933 patients with different mental disorders, of which 3002 patients have schizophrenia and 3931 have other mental disorders. A total of 11,884 admission records corresponds to the 6933 patients, of which 5968 records belong to patients with schizophrenia and 5916 records belong to patients with other mental disorders. The most representative gender in the data set is male with 55.9% (6639 of 11,884 records), while females represent 44.14% (5245 of 11,884 records). The age of the patients is between 13 and 97 years.

The data are anonymized and follow the international coding standard for diseases ICD-9 [52]. The database collects minimum data set information such as the hospital to which the patient belongs, age, gender, dates of admission and discharge from the hospital, days of stay, the main diagnostic diseases of psychiatric type, secondary diagnoses, and complementary therapies to each medical diagnosis. The procedures applied in the data set have been carried out, guaranteeing the patient’s privacy and security.

### 2.2. System Architecture

**Figure 1:**
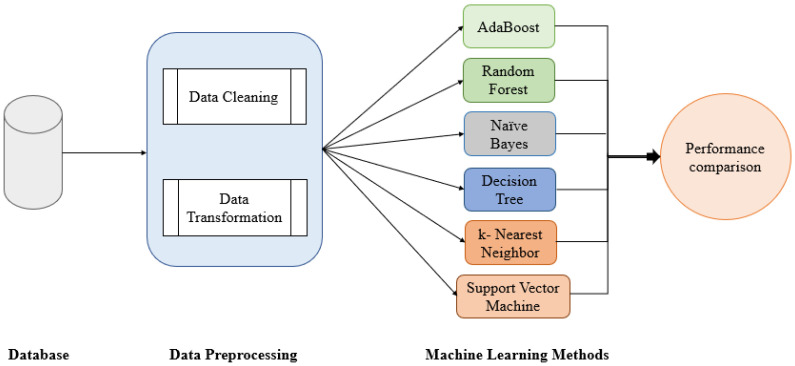
Flow diagram of the study. The diagram shows the first phase of pre-processing the database. Subsequently, the machine learning algorithms described are applied to the pre-processed dataset. In the final phase, the performance metrics obtained from the algorithms are compared.

The proposed predictive model includes data processing and feature selection before the application of machine learning techniques (see Figure 1).

The data pre-processing phase includes cleaning and data processing. For the development of this phase, the programming environment R version 3.6.3 was used, applying the dplyr [53] and tidyr [54] packages. The authors have performed a general exploratory analysis considering the type of data. In this phase, an analysis of nulls, zeros, and atypical values has been carried out, where null values, double white spaces, and special characters have been eliminated to avoid false classifications according to [55]. Furthermore, data normalization has been used with an interval (0–1) as suggested by [56]. Finally, a longitudinal and data coherence analysis was performed.

The original data set has a total of 27 different variables for the prediction of hospitalized patients with schizophrenia. The authors have classified them to obtain the best predictive features. For the selection of features, we have used the Gain Information, Gain Ratio, Gini, X^2^, and ReliefF methods. These techniques have been used to analyze the relationship between each input variable and the target variable according to previous research activities [15,29]. The threshold value chosen was Gain Information ≥ 0.009, Gain Ratio ≥ 0.005, and Gini ≥ 0.007. Consequently, the input variables with better scores have been used in the model. Table 1 shows the classification and metrics of the subset of features obtained.

Once the selection of features with the supervised filter method has been carried out, 8 predictive variables are chosen to use in the study. The Diag_Sec02_Code, Diag_Sec03_Code, Diag_Sec04_Code, Diag_Sec05_Code, and Diag_Sec06_Code features contain the disease codes established by ICD-9 [52], which correspond to the diagnoses of admitted patients. Considering the clinical aspect of these 5 features, each one of them includes the following classification of diseases: (1) mental health disorders (Codes 290.0-319), (2) nervous system (Codes 323.9-389.9), (3) infectious diseases (Codes 041.4-138), (4) neoplasias (Codes 141-239.6), (5) circulatory system (Codes 394.1-459.81), (6) endocrine diseases (Codes 240-279.11), (7) respiratory system (Codes 461.1-519.8), (8) digestive system (Codes 521.09-579.8), (9) genitourinary system (Codes 584.8-626.6), (10) blood diseases (Codes 280-289.81), (11) skin and subcutaneous tissue (Codes 681.02-709.01), (12) lesion and poisoning (Codes 801.30-999.2), (13) congenital anomaly (Codes 743.61-759.89), and (14) arthropathies and related disorders (Codes 710.1-737.39). Stays_Days corresponds to days of stay in the healthcare complex, and we have included personal data such as age and gender variables. The Diag_Schizophrenia variable is the target class. These variables are used in Decision Tree, AdaBoost, Random Forest, k-NN, Naïve Bayes, and SVM algorithms to predict hospitalized patients with schizophrenia. For the implementation of the model, a MacBook Pro (2018) running on a macOS Catalina version 10.15.3 operating system has been used to perform predictive analysis, develop the machine learning models, and data visualization.

#### Algorithms of Machine Learning

In the study, we evaluated different machine learning algorithms such as Random Forest, Naïve Bayes, SVM, Decision Tree, AdaBoost, and kNN. Table 2 shows the parameters of the machine learning algorithms used in the study.

Regarding the Decision Tree algorithm, the model induces a binary tree, the minimum number of instances used on the leaves is two instances, the model does not split subsets smaller than five, the maximum depth is 100, and the algorithm stops dividing when most nodes reach 95%. The AdaBoost algorithm is an iterative ensemble method that builds a strong classifier by combining multiple low-performing classifiers. The base estimator that we used to train the model was Decision Tree with 50 estimators.

The Naïve Bayes algorithm uses the adjustment parameter usekernel with a value False to assume a Gaussian density distribution, the correction factor Laplace (fL) is 0, and the parameter adjust was kept constant at a value of 0. For the k-NN algorithm, the number of neighbors is 5 and the distance metric is Euclidean, while for SVM we use C = 1.0 and sigma = 0.5, radial basis function as a kernel parameter, numerical tolerance = 0.001, and iteration limit = 100.

The Random Forest algorithm is described as a joint learning technique since it combines results from multiple decision trees, returning a single prediction. Random Forest models are made up of a set of individual decision trees, each trained on a different sample of the training data, generated by bootstrapping. Prediction of a new observation is obtained by adding the predictions of all the individual trees that make up the model [49,57]. The parameters used are 10 trees, the maximum number of features considered is unlimited, there is no replicable training, the maximum depth of the tree is unlimited, and the nodes stop dividing into five maximum instances (See Table 2).

The pseudocode of Random Forest algorithm is presented as follows:

1. Select “*k*” features from total “*m*” features at random where *k* < *m*;
2. Calculate the node “*d*” through the better split point among “*k*” feature;
3. Split the node into child nodes through the better split;
4. While “l” number of nodes has been done, redo steps 1 to 3;
5. Create forest by doing steps 1 to 4 by “*n*” times to build “*n*” set of trees.

**Figure 2:**
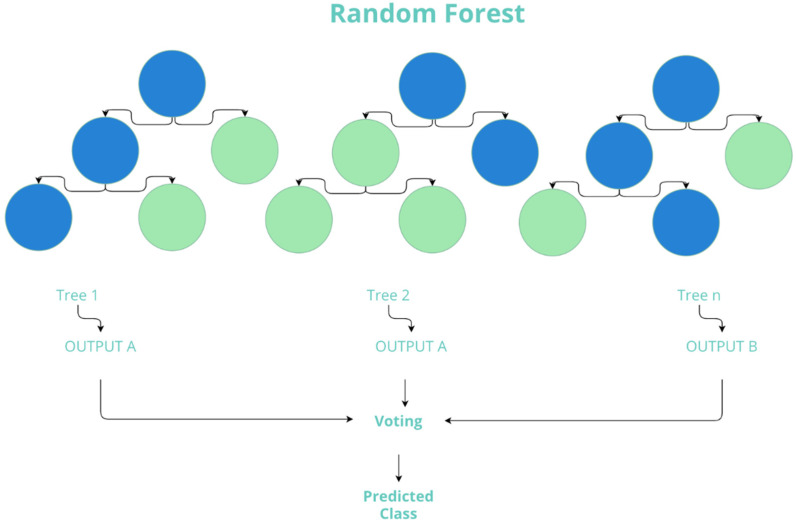
Diagram of the Random Forest algorithm. The algorithm generates multiple trees; in the figure, each set represents a tree. Each tree classifies a class, resulting in the class with the highest number of votes.

Figure 2 shows the visual diagram of the Random Forest algorithm.

For the evaluation of the predictive model, we have followed the approach of other studies [33,58], using a k-fold stratified cross-validation procedure [35,38]. To avoid the overfitting problem, the authors have assigned a value to *k* = 10. This validation method divides the entire data into 9 training folds and 1 validation fold. Each fold is used once as the test set, while the remaining folds are used for training. Consequently, all the data is used both for training and testing. The results obtained with each algorithm are compared with each other considering different machine learning performance metrics: accuracy, precision, F1-score, AUC, and recall.

## 3. Results

### 3.1. Data Analysis

The study includes a total of 11,884 records corresponding to 6933 hospitalized patients with several mental health disorders. Analysis of the clinical data shows significant differences regarding the gender of hospitalized patient records with schizophrenia and without the disease (See Table 3). In the records of schizophrenia, men represent 71.0% (4238 of 5968 records), being the most affected by this psychiatric disorder, compared to women who represent 29.0% (1730 of 5968 records). On the other hand, in records without schizophrenia, men represent 40.6% (2403 of 3513 records) and women represent 59.4% (3513 of 5916 records). The group from 31 to 60 years is the most representative age of the data set and represents 64.9% (7717 of 11,884 records). Regarding the days of stay and age mean, both groups of records have similar behavior. Records with schizophrenia show a mean age of 43 years and a mean stay of 17 days, while the records with other disorders show a mean age of 49 years and 14 days of stay.

The Diag_Sec02_Code is the second most relevant variable in the study according to the ranking of features in Table 1. The analysis of the main diagnoses of this variable shows different disorders in both groups of records. Records with schizophrenia show 473 records of non-compliance with medical treatment, 353 records of tobacco abuse disorders, 229 of a family record of psychiatric disease, 200 records of continuous cannabis abuse, and 145 records of alcohol abuse. The diagnoses of patient records without schizophrenia present 687 records with dysthymic disorder, 265 records with personality disorder, 177 records with neom arterial hypertension, 167 records with personality histrionic disorder, and 162 records with neom psychosis.

### 3.2. Model Evaluation

The authors have used the Random Forest, AdaBoost, Naïve Bayes, k-NN, Decision Tree, and SVM algorithms to predict hospitalized patients with schizophrenia. The evaluation of the predictive model was performed using a 10-fold stratified cross-validation. The performance results of the methods used are shown in Table 4. The results when identifying schizophrenia in hospitalized patients against each of these groups separately are presented in Supplementary Materials Tables S1 and S2.

Consequently, Random Forest presents the best accuracy with 72.7%, as well as the rest of the metrics. AdaBoost and Decision Tree showed 70.8% and 68.2% accuracy, respectively. Naïve Bayes reports 67.0% accuracy, k-NN with 67.7% accuracy, and SVM with the lowest value of accuracy at 65.7%. Naïve Bayes and k-NN algorithms present better results in terms of AUC compared to Decision Tree; however, Decision Tree improves in terms of accuracy, precision, F1-score, and recall.

**Figure 3:**
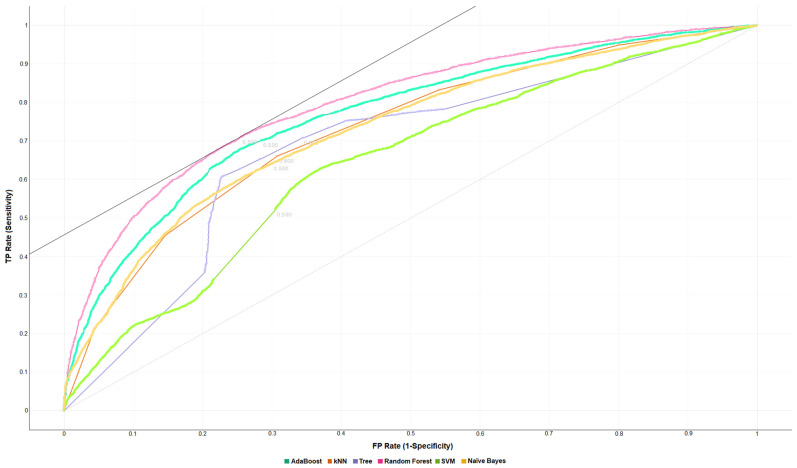
ROC curve for target = 0 with FP = 500, FN = 500, and target probability = 50.0%. The graph shows the ROC curves created by the false positive and false negative values with 10-folds stratified cross-validation. Each ROC curve is represented by a different color (See legend). The Random Forest algorithm shows the best value of AUC= 0.796 (See Supplementary Materials Table S1) for class 0 (records of non-schizophrenia).

**Figure 4:**
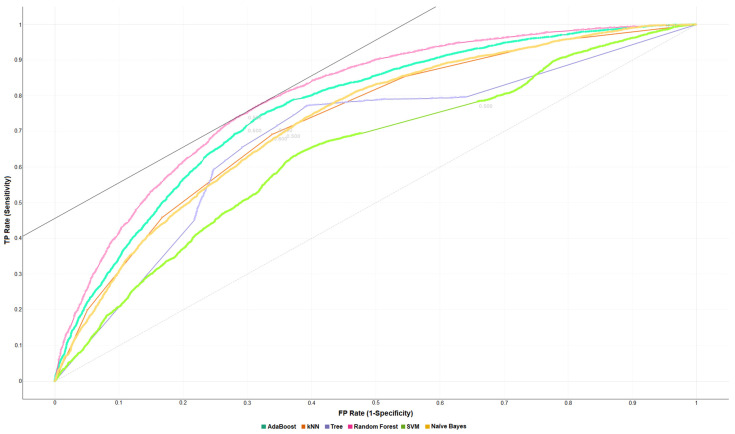
ROC curve for target = 1 with FP = 500, FN = 500, and target probability = 50.0%. The graph shows the ROC curves created by the false positive and false negative values with 10-folds stratified cross-validation. Each ROC curve is represented by a different color (See legend). The Random Forest algorithm shows the best value of AUC= 0.796 (See Supplementary Materials Table S2) for class 1 (records of schizophrenia).

The ROC curves (receiver operating characteristic curve) shown in Figure 3 and Figure 4 show graphs of sensitivity vs. 1-specificity through different cut points.

The authors evaluated for target = 0 and target = 1 with False Positive (FP) = 500, False Negative (FN) = 500, and probability of target = 50.0%. In this way, it is possible to visually evaluate the general performance of each classification algorithm to be evaluated. AUC under the ROC curve provides a normalized mean performance of the classifier, considering the entire range of output decision thresholds in the plane of specificity-sensitivity.

Considering the presented ROC curves created for the 10-fold stratified cross-validation, the authors obtained an AUC value of 0.796, 0.765, 0.682, 0.729, 0.729, and 0.641 for Random Forest, AdaBoost, Decision Tree, k-NN, Naïve Bayes, and SVM, respectively. These values recommend the Random Forest algorithm as the best normalized average classifier performance, with an AUC value much higher than the other algorithms. Therefore, when comparing the ROC curves shown in Figure 3 and Figure 4, the sensitivity and specificity values are balanced, and the algorithms discriminate between hospitalized patients with schizophrenia and without the disorder with approximately the same probability.

The applied method is compared with some studies available in the literature that use similar techniques (See Table 5).

## 4. Discussion

The diagnosis of mental disorders such as schizophrenia is the first step in a set of actions that are selected to save patients’ lives or improve their health [59]. People with this disorder tend to be hospitalized frequently and have high rates of disability, imposing an economic cost on a general level. Therefore, from the viewpoint of our study, the implementation of predictive models in medical systems can be a helpful tool in the prevention of hospitalizations of patients with schizophrenia in the region of Castilla and León.

Multiple research studies use EEG and functional magnetic resonance imaging (fMRI) to identify patients with schizophrenia using machine learning techniques [19,41,60,61]. Other studies consider symptoms, cognitive functions, and non-verbal signals to explore the feasibility of automatic interview transcripts to classify schizophrenic patients [62,63].

In our study, the most relevant variables considered according to the ranking of features in Table 1 are Gender, Diag_Sec02_Code, and Age. Regarding the clinical aspect and the behavior of these variables, the gender of hospitalized patient records with schizophrenia is determinant. Men have a higher risk of suffering acute hospital admissions by the disease in the age range from 28 to 50 years compared to women. In the secondary diagnoses of these patients, they mainly present disorders of tobacco, alcohol, and cannabis abuse, determining substance abuse as a risk factor. The hospitalized patient records without schizophrenia do not show a significant difference regarding the patient’s gender and age. The main physical diagnoses in this group are personality disorders and arterial hypertension, which are not shown as the main risk factors for hospitalized patients with schizophrenia in this study.

The classification methods used have a predictive accuracy of 65.7–72.7%, in terms of predicting hospitalized patients with schizophrenia (See Table 4). When comparing the performance metrics of the six machine learning methods, Random Forest is recommended as the best performance accuracy with 72.7%.

In the study proposed by the authors of [35], the classification of suicides in patients with schizophrenia presented an accuracy rate of 66.0% with an AUC = 0.67 and AUC = 0.70 for Random Forest and SVM, respectively, considering a sample of 345 patients. In [38], the authors diagnosed schizophrenia using four machine learning algorithms. The study presented an accuracy of 58.2–68.6% with an AUC of 0.63–0.68, with Random Forest obtaining the highest values, and SVM obtaining the lowest value.

The results presented in this research using machine learning algorithms show a reliable approach to predict hospitalized patients with schizophrenia in public hospitals in the Castilla and León region. However, the proposed study has several limitations. There is a relevant limitation regarding the significance features available in the data set used. From the 27 variables of the original data set, the authors have selected only 8, for a sample of 11,884 records. The selection of 8 features has the advantages of using less computational requirements in the development of the model and fast processing of the data collection. Furthermore, the age range of the participants in this study is broad. The authors aim to perform new experiments using patients with a strict age range to understand the impact of age in predicting schizophrenia.

Comparing the data set used in this study with other studies presented in the literature, the number of features (N = 8) used is inferior. In the study [37], the authors with a sample of 20 features obtained an average classification accuracy of the combined feature set of 78.2% using SVM. In [36] with a sample of 204/410 features, the authors obtained accuracy values of 86.04–90.69% using SVM, ANN, Random Forest, and Naïve Bayes algorithms. By reducing the number of features to 11, the values decrease to 82.55–83.72%. Therefore, the authors consider that it is necessary to increase the number of features to obtain metrics with higher values; however, this will require more computing resources and time for data collection. In general, in machine learning models, the sample size and predictor variables influence the training and effective validation of the predictions [49]. In our study, the classification of hospitalized patients with schizophrenia is based mainly on disease diagnostic features. If we compare our attributes with those used in studies such as [19,37,41], which focused on the prediction of schizophrenia using EEG and fMRI, we obtain performance metrics with a lower value. If we compare our attributes with the study [38], our model performance is higher. Therefore, the inclusion of new attributes related to neuroimaging can affect the performance of our models and allow improvement in patient-focused treatments. The database used in the study contains hospitalized patients with schizophrenia and other mental health diseases as the main diagnostic. Similar studies found in the literature show results between healthy and schizophrenia patients; this notable difference becomes a limitation of the study since predictive models cannot be developed using records of healthy patients. Furthermore, it is necessary to mention that in the study we only evaluated patients who are hospitalized, and people with schizophrenia from that region who are not hospitalized have not been included. Therefore, the results obtained are not generalizable to the entire population with schizophrenia in the Castilla and León region. Another limitation of our study is that we only applied six machine learning algorithms based on previous studies available in the literature [27,35,50]. However, it is necessary to emphasize that we presented the model parameters when most related studies did not provide this information, which was necessary to reproduce the results. In the future, the authors aim to use different machine learning techniques for improved accuracy.

The use of predictive models can be applied in different types of hospitals (private and/or public) to predict hospitalized patients with schizophrenia. These models can help in the hospital management of this type of disorder. Furthermore, the predictive model approach would be a useful tool for preventing hospitalizations of patients with schizophrenia in this region, reducing hospitalization costs.

## 5. Conclusions

This paper presents the development of prediction models of hospitalized patients with schizophrenia using k-NN, Decision Tree, SVM, Naïve Bayes, Random Forest, and AdaBoost machine learning algorithms and compares their system performance. The study identifies the AdaBoost (accuracy = 70.8% and AUC = 0.765) and Random Forest (accuracy = 72.7% and AUC = 0.796) ensemble algorithms as the best at classifying hospitalized patients with schizophrenia in this population. These algorithms present a higher value in their performance metrics than the SVM (accuracy = 65.7% and AUC = 0.641) algorithm. The results obtained suggest that the use of algorithms such as Random Forest can help in the hospital management of this type of disorder. The predictive modelling approach would be a useful tool in the prevention of hospitalizations of patients with schizophrenia in this region. Therefore, based on future lines, we will focus on the prediction of risk factors in hospitalized patients with schizophrenia and the prediction of readmissions in acute units of each health complex.

## Figures and Tables

**Table 1:** Metrics and ranking of the features.

Variables	Information Gain	Gain Ratio	Gini	X^2^	ReliefF
Diag_Sec02_Code	0.047	0.023	0.032	128.578	0.012
Diag_Sec03_Code	0.014	0.007	0.010	0.044	0.006
Diag_Sec04_Code	0.011	0.006	0.008	91.753	0.004
Diag_Sec05_Code	0.016	0.010	0.011	269.514	0.003
Diag_Sec06_Code	0.014	0.012	0.010	331.083	0.019
Stays_Days	0.009	0.005	0.007	128.946	−0.0003
Age	0.025	0.012	0.017	310.541	0.012
Gender	0.069	0.070	0.047	623.212	-
Admission_Type	0.0004	0.002	0.0003	0.238	−0.002
Proc_Ppal_Code	0.005	0.003	0.004	49.338	0.015
Proc_Sec02_Code	0.005	0.003	0.003	100.140	−0.014
Proc_Sec03_Code	0.004	0.004	0.003	96.206	−0.005

**Table 2:** Parameters of the machine learning algorithms used in the study.

Algorithms	Parameters
Random Forest	Number of trees = 10
	Maximum number of considered features: unlimited
	Maximum tree depth: unlimited
	Stop splitting nodes with maximum instances = 5
AdaBoost	Base estimator: tree
	Number of estimators = 50
Decision Tree	Minimum number of instances in leaves = 2
	Minimum number of instances in internal nodes = 5
	Maximum depth = 100
kNN	Number of neighbours = 5
	Distance metric: Euclidean
	Weight: Uniform
Naïve Bayes	fL = 0
	usekernel: False
	adjust = 0
SVM	C = 1.0
	sigma = 0.5
	Numerical tolerance = 0.001
	Iteration limit = 100

**Table 3:** Analysis of clinical data.

Variables	Total (N = 11,884 Admission Records)	
	*n* = 5968 Records Schizophrenia	*n* = 5916 Records Non-Schizophrenia
Gender (%)		
Male	71.0	40.6
Female	29.0	59.4
Age, mean (years)	43	49
<18 years	10	36
18–30 years	1048	737
31–45 years	2493	1756
46–60 years	1624	1844
>60 years	793	1543
Days of stay, mean (days)	17	14
Main diagnoses of the predictive variable Diag_Sec02_Code for records with schizophrenia		
Non-compliance with medical treatment	473	130
Tobacco abuse disorders	353	111
Family record of psychiatric disease	229	118
Abuse of continuous cannabis	200	70
Alcohol abuse	159	86
Main diagnoses of the predictive variable Diag_Sec02_Code for records without schizophrenia		
Dysthymic disorder	19	687
Personality disorder	75	265
Neom arterial hypertension	140	177
Personality histrionic disorder	4	167
Psychosis	40	162

**Table 4:** Performance metrics applying 10-fold stratified cross-validation.

Algorithms	AUC	Accuracy	Precision	F1-Score	Recall
Random Forest	0.796	0.727	0.728	0.727	0.727
AdaBoost	0.765	0.708	0.708	0.708	0.708
Decision Tree	0.682	0.682	0.682	0.681	0.681
k-NN	0.729	0.677	0.676	0.676	0.676
Naïve Bayes	0.729	0.670	0.671	0.669	0.670
SVM	0.641	0.657	0.657	0.657	0.657

**Table 5:** Comparison of results obtained with other studies.

Reference	Method	Validation	Dataset	AUC	Accuracy (%)
[35]	Random Forest	Cross-Validation k = 10	N = 345 patients	0.67	66.00
[36]	Random Forest Naïve Bayes SVM	Cross-Validation k = 10	N = 86 patients	-	90.69
[37]	SVM	Leave-One-Out Cross-Validation (LOOCV)	N = 68 patients	-	78.24
[38]	Random Forest	Cross-Validation k = 10	N = 72 patients	0.68	68.60
[39]	Random Forest	Cross-Validation	N = 466 patients	-	85.10
Our study	Random Forest	Cross-Validation k = 10	N = 6933 patients	0.79	72.74

## Data Availability

Third party data and restrictions apply to the availability of these data. The data were obtained from the Junta de Castilla y León and the Hospital of Zamora; therefore, they are available with the permission of both institutions.

## Supplementary Materials

The following supporting information can be downloaded at: https://www.mdpi.com/article/10.3390/s22072517/s1, Table S1: Scores with target = 0; Table S2: Scores with target = 1.
