# Supplementary material for: Comparison of Machine Learning Algorithms in the Prediction of Hospitalized Patients with Schizophrenia
Source: Sensors (Basel). 2022 Mar 25;22(7):2517. doi: 10.3390/s22072517 (PMC9003328; doi:10.3390/s22072517)
Supplement: Supplementary file 1 [file sensors-22-02517-s001.zip › sensors-1641683-supplementary.pdf]

Supplementary Materials

Table S1. Scores with target=0

Sampling type: Stratified 10-fold Cross validation
Target class: 0 - non-schizophrenia

Model	AUC	CA	F1	Precision	Recall
Random Forest	0.7959301837306404	0.7273645237293841	0.7216494845360824	0.7337526205450734	0.7099391480730223
AdaBoost	0.7680043282662455	0.7081790642881185	0.7052524222335544	0.7092307692307692	0.7013184584178499
Tree	0.6819721514995212	0.6817569841804106	0.6888267237123581	0.6710484129528695	0.7075726842461122
kNN	0.7283872029760143	0.6765398855604173	0.6704389574759945	0.6802366040361865	0.6609195402298851
Naive Bayes	0.7286422776908836	0.6702288791652642	0.6592470219980873	0.6787824529991048	0.6408045977011494
SVM	0.6622895822275844	0.6572702793672164	0.6590775927010964	0.6527938981926712	0.6654834347532116

Table S2. Scores with target=1

Sampling type: Stratified 10-fold Cross validation
Target class: 1 - schizophrenia

Model	AUC	CA	F1	Precision	Recall
Random Forest	0.7959471905356531	0.7273645237293841	0.7328496042216359	0.7214285714285714	0.7446380697050938
AdaBoost	0.7680098545673701	0.7081790642881185	0.7110481586402266	0.707159429897249	0.7149798927613941
Tree	0.6819721514995212	0.6817569841804106	0.674358532805235	0.6935883811547998	0.6561662198391421
kNN	0.7283872029760143	0.6765398855604173	0.6824190350297423	0.6730769230769231	0.6920241286863271
Naive Bayes	0.7286422776908836	0.6702288791652642	0.6805249857340833	0.6626448642641689	0.699396782841823
SVM	0.6622895822275844	0.6572702793672164	0.6554437018864732	0.6618827951477875	0.6491286863270778
